# The Learn Together programme (part B): evaluating co-designed guidance to support patient and family involvement in patient safety incident investigations

**DOI:** 10.3389/frhs.2025.1520816

**Published:** 2025-04-22

**Authors:** Lauren Ramsey, Justin Waring, Laura Sheard, Daisy Halligan, Siobhan McHugh, Ruth Simms-Ellis, Joe Langley, Jenni Murray, Olivia Rogerson, Jane K. O'Hara

**Affiliations:** ^1^ Yorkshire and Humber Patient Safety Research Collaboration, Bradford Institute for Health Research, Bradford, United Kingdom; ^2^ School of Social Sciences and Humanities Loughborough University, Loughborough, United Kingdom; ^3^ York Trials Unit, University of York, York, United Kingdom; ^4^ School of Humanities and Social Sciences, Leeds Beckett University, Leeds, United Kingdom; ^5^ School of Psychology, University of Leeds, Leeds, United Kingdom; ^6^ Lab4Living, Sheffield Hallam University, Sheffield, United Kingdom; ^7^ The Healthcare Improvement Studies (THIS) Institute, University of Cambridge, Cambridge, United Kingdom

**Keywords:** patient safety, patient involvement, healthcare harm, safety investigations, healthcare litigation, qualitative research

## Abstract

**Background:**

Expectations of patient and family involvement in investigations of healthcare harm are becoming conventional. Nonetheless, *how* people should be involved, is less clear. Therefore, the “Learn Together” guidance was co-designed, aiming to provide practical and emotional support to investigators, patients and families.

**Aim:**

To longitudinally evaluate use of the Learn Together guidance in practice—designed to support patient and family involvement in investigations of healthcare harm.

**Methods:**

A 15-month process evaluation took place across five sites, following 29 investigations in which the Learn Together guidance was used. Sites comprised two mental health and two physical health hospital Trusts, and an independent maternity investigatory body in England. Longitudinally, 127 interviews were conducted with investigators, patients, families, staff, and management. Interview and observational data were synthesized using Pen Portraits and analyzed using multi-case thematic analysis.

**Findings:**

The guidance supported the systematic involvement of patients and families in investigations of healthcare harm and informed them how, why, and when to be involved across settings. However, within hospital Trusts, investigators often had to conduct “pre-investigations” to source appropriate details of people to contact, juggle ethical dilemmas of involving vs. re-traumatizing, and work within contexts of unclear organizational processes and responsibilities. These issues were largely circumvented when investigations were conducted by an independent body, due to better established processes, infrastructure and resources, however independence did introduce challenge to the rebuilding of relationships between families and the hospital Trust. Across settings, the involvement of patients and families fluctuated over time and sharing a draft investigation report marked an important part of the process—perhaps symbolic of organizational ethos surrounding involvement. This was made particularly difficult within hospital Trusts, as investigators often had to navigate systemic barriers alone. Organizational learning was also a challenge across settings.

**Conclusions:**

Investigations of healthcare harm are complex, relational processes that have the potential to either repair, or compound harm. The Learn Together guidance helped to support patient and family involvement and the evaluation led to further revisions, to better inform and support patients, families and investigators in ways that meet their needs (https://learn-together.org.uk). In particular, the five-stage process is designed to center the needs of patients and families to be heard, and their experiences dignified, before moving to address organizational needs for learning and improvement. However, as a healthcare system, we call for more formal recognition, support and training for the complex challenges investigators face—beyond clinical skills, as well as the appropriate and flexible infrastructure to enable a receptive organizational culture and context for meaningful patient and family involvement.

## 1 Introduction

Around 10,000 incidents of avoidable death happen in the United Kingdom (UK) healthcare system annually (1), emblematic of a global burden of harm. Steadily the value of patient and family involvement after healthcare harm has been acknowledged. Over two decades ago, Vincent and Coulter (2) advocated for the active and expansive roles which patients and their families should play in patient safety policies and procedures, including in the aftermath of harm. This once radical set of ideas, was bolstered by a history of patient activism (3) and was furthered by evidence highlighting the important perspective of patients [e.g., (4–7)]. In addition, there have been repeated accounts of the undoubtedly harmful effects patients experience when not listened to [e.g., (8, 9)]. The term “compounded harm” was coined to refer not to original incident related harms, but additional harms created by processes that follow due to “neglecting to appreciate and respond to human impacts” (10). A typology of compounded harms identified that people can be left feeling powerless, inconsequential, manipulated, abandoned, de-humanised and disoriented as a result (11). Therefore, whilst it may not be possible to achieve zero harm in a complex, dynamic healthcare system, it is important that the health service proceeds in the wake of safety events in ways that do not further traumatize those affected. The Parliamentary Health Services Ombudsmen (PHSO) claimed that the concept of compounded harm has long been neglected (12).

In recognition of this issue, global policy has highlighted expectations of patient and family involvement. For example, the Joint Commission has required healthcare providers in America to disclose “unanticipated outcomes” of care to patients since 2001. Following well-documented care failings (13), since 2014, staff in the UK NHS are expected to tell patients when something has gone wrong, apologize and offer appropriate remedy as per the Duty of Candour. Across the world, this is underpinned by World Healthcare Organizations' action plan (14), aiming to “establish the principle and practice of openness and transparency throughout health care, including through patient safety incident disclosure to patients and families”. The Patient Safety Incident Response Framework (PSIRF), coming into effect in 2022 in the UK, enlarged on this, and proposed somewhat detailed guidance about the importance of compassionately engaging and involving patients and their families after harm (15).

Nonetheless, *how* patients and families should be involved in practice, is much less clear. Tested in America are Communication Resolution Programmes (CRP's) [e.g., (16)], the Improving Post-Event Analysis and Communication Together (IMPACT) tool (17), the Disclosure, Apology and Offer Model (DA&O) (18) and the Recognize, Respond and Resolve (3R's) approach (19), all of which emphasize the need for honest and open communication with patients and their families. In addition, Open Disclosure has been tried in Australia and the UK [e.g., (20, 21)], and Next-of-kin involvement has been implemented in Norway (22, 23). Ramsey et al. (24), reviewed the literature regarding these interventions, as well as the wider empirical evidence, and found that fear of litigation remained a significant barrier to involvement, even when interventions were designed to circumvent it. It was also particularly important that any responses to healthcare harm attended to both clinical and emotional aspects of care (24).

**Figure 1:**
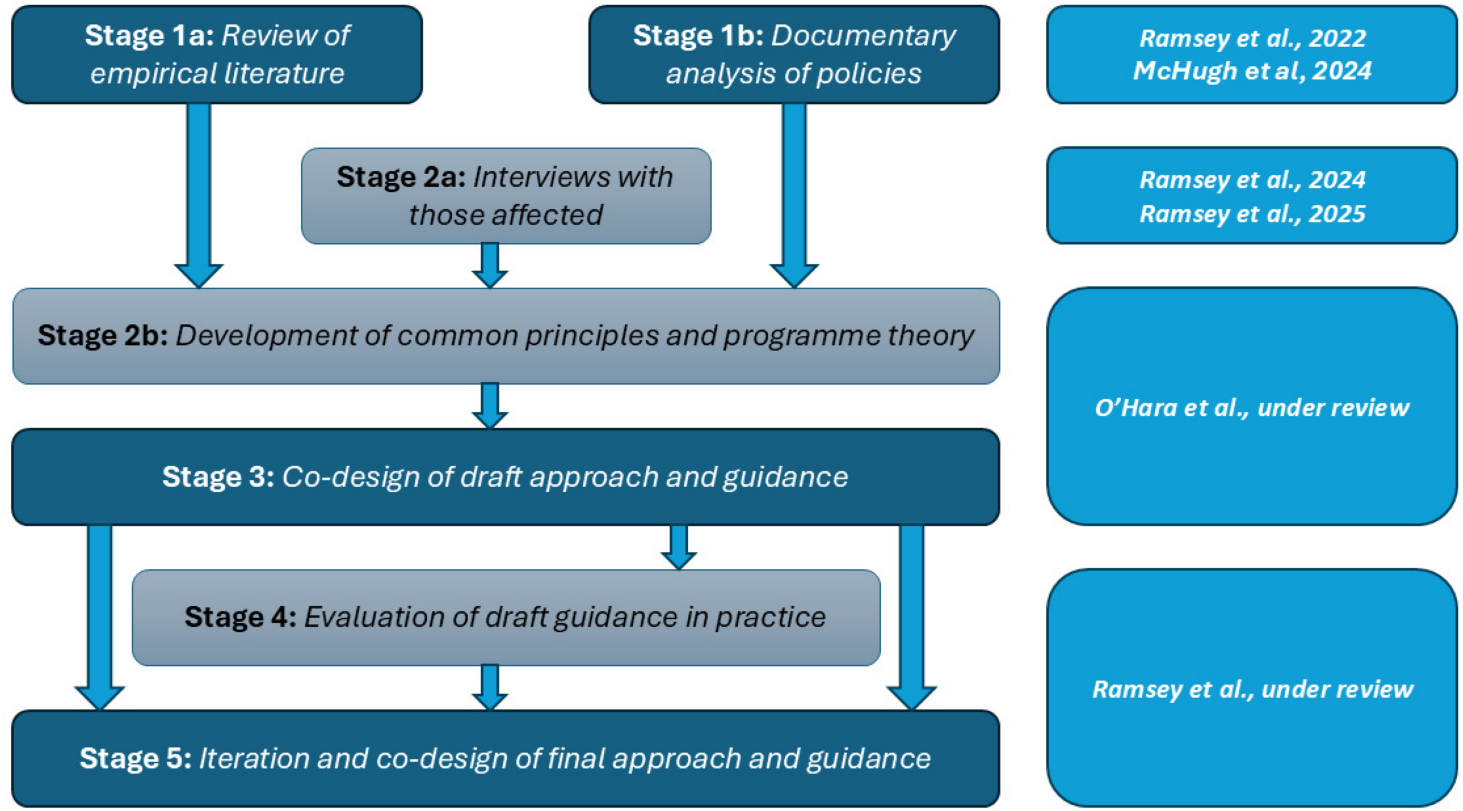
Overview of the learn together programme (https://learn-together.org.UK).

The Learn Together programme brought together this existing empirical evidence (24), findings from a review of local hospital Trust policy in England (25) and interviews with those affected (11, 26) to develop common principles for involvement and an-in-depth co-design phase (27, 28). From this synthesis, the “Learn Together guidance” was developed (27, 28). This aimed to bridge the gap in resources, especially within the English NHS, to provide investigators, patients and families with practical and emotional support following healthcare harm. However, it was yet to be evaluated in practice. Therefore, we undertook an evaluation which formed stages 4 and 5 of the Learn Together programme—5 years of research funded by the National Institute for Health and Care Research (NIHR) (https://learn-together.org.uk). Figure 1 provides an overview of the programme. Specifically, this study aimed to evaluate the co-designed Learn Together guidance and explored how it influenced processes.

## 2 Methods

This ethnographic study received favourable ethical approval in October 2021 (REC ref 21/WA/0287) and draws upon data collected between January 2022 and March 2023.

### 2.1 The Learn Together guidance

The Learn Together guidance was co-designed by a community of >50 people including patients, families, staff, investigators, managers, policy makers, lawyers and academics (see Box 1). The co-design process, management of ideas, outcomes, and challenges is detailed by O'Hara et al. (27, 28). The guidance was designed to be used alongside training and provide investigators, patients and families with information and support to be involved in investigations, and to make the process as easy and as meaningful as possible.

Box 1 **The learn together guidance version 1. Reproduced with permission from “Learn Together Resources” by Jane O'Hara, licensed under CC BY 4.0.**

Investigator guidance (27, 28) 
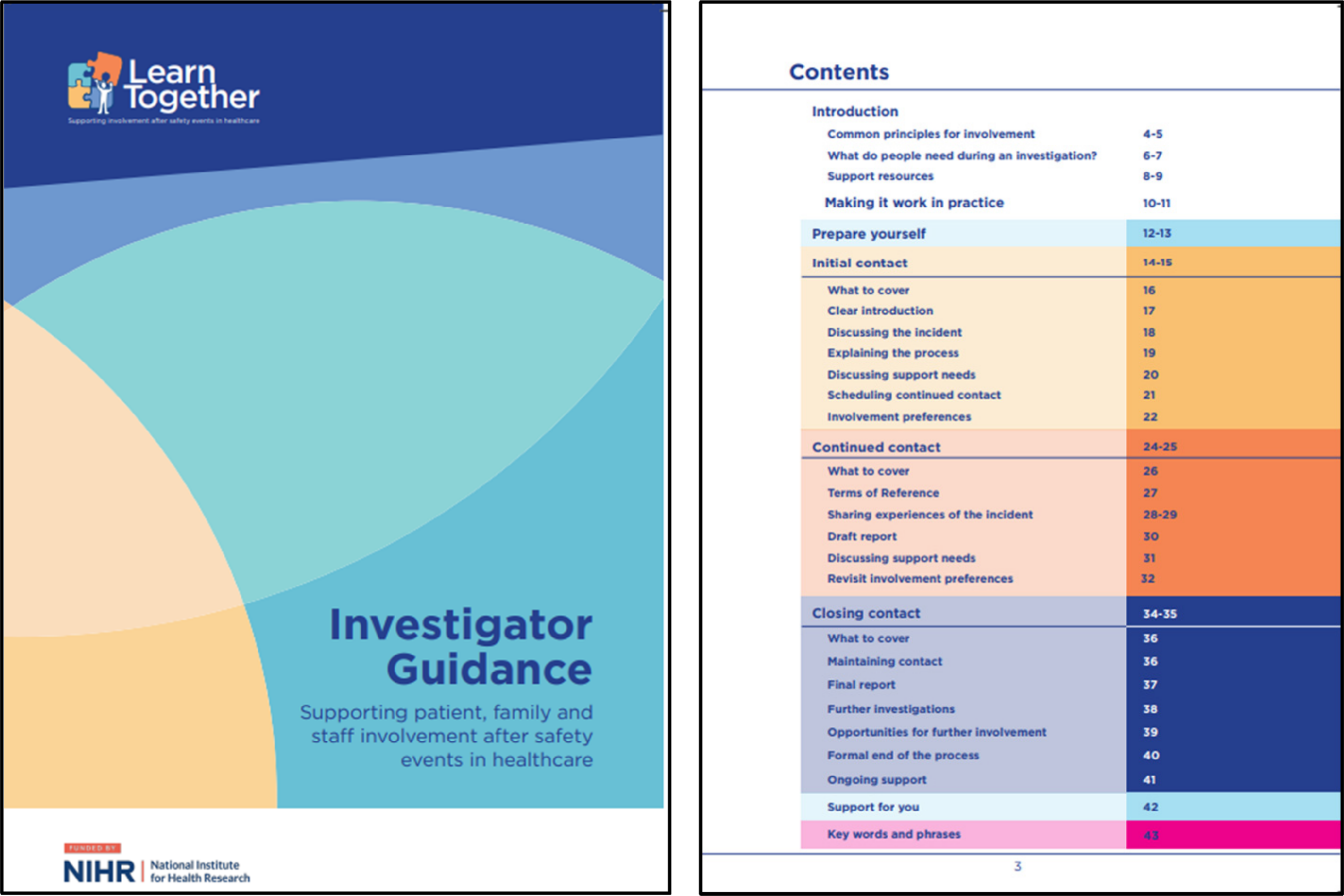
 The Investigator guidance booklet provided an introduction of key background information including the common principles for involvement, what people need during an investigation and sources of support, as well as information about how to make this work for them in practice. Investigators were guided through patient and family involvement in investigations at each stage, broken down into subsections: “prepare yourself”, “initial contact”, “continued contact”, “closing contact” and “support for you”. Patient and Family Guidance (27, 28) 
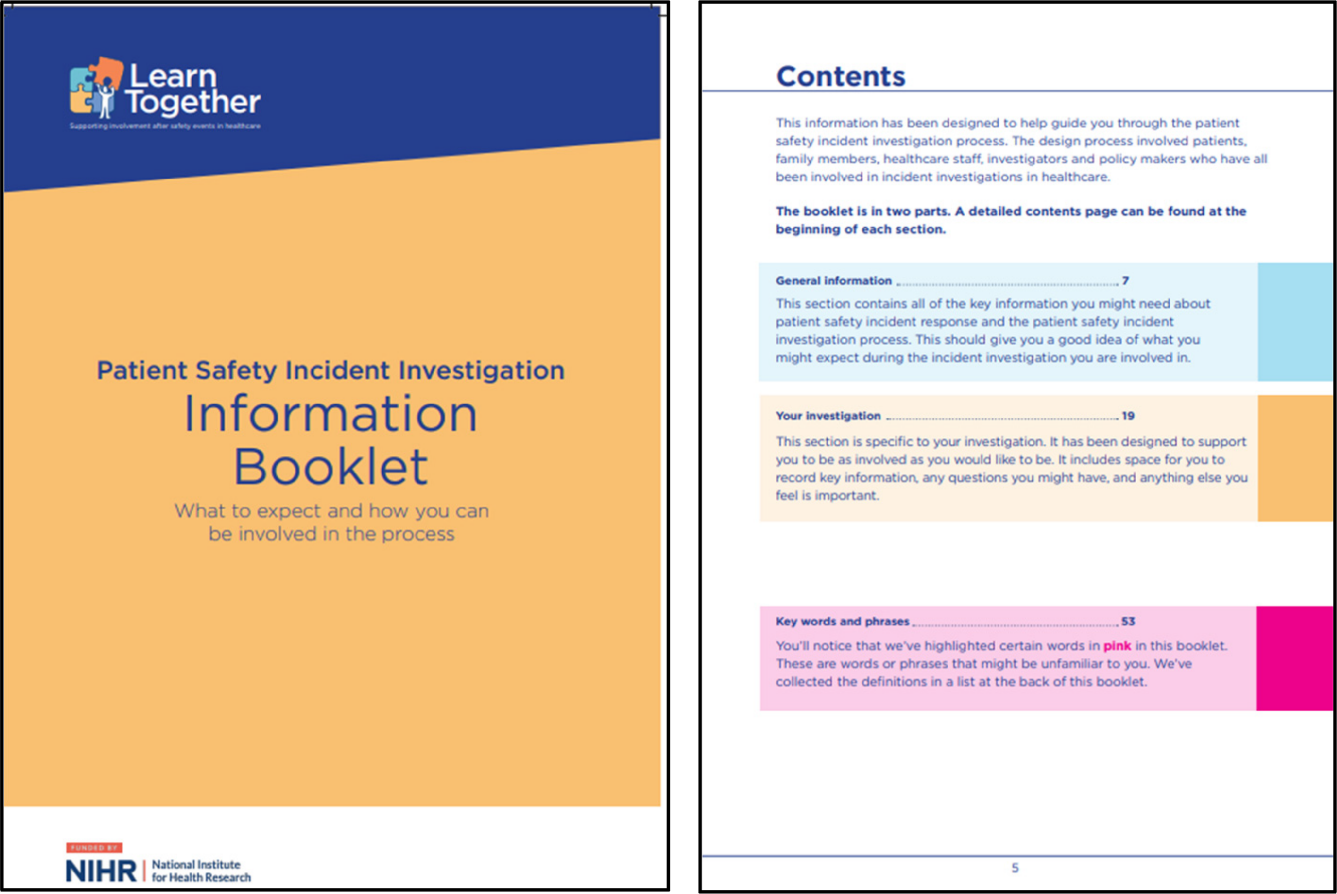
 The patient and family guidance provided information designed to support patients and their families through the process. The content of the booklet was broken down into two key parts. First, “general information”, which was designed to help people know what to expect during the serious incident investigation process and prepare them to engage from an informed position. Second, “your investigation”, which was designed to support people to be involved as much as they would like to be, with reflective space to record information, questions and any other information that may feel important. *Other documents:* *1. Investigation record* *2a. National investigatory body—investigator support* *2b. National investigatory body—patient and family reflective booklet*

### 2.2 Focused-ethnographic approach

A focussed-ethnographic approach (29) to process evaluation was led by four field researchers (LR, DH, SMcH, OR) and took place in three settings across five sites in England comprising: (i) two mental health hospital Trusts (MH1, MH2), (ii) two physical health hospital Trusts (PH1, PH2), and (iii) a national, independent investigatory body (IND). The guidance was used within 29 investigations across sites. Fieldwork comprised 127 longitudinal interviews with key stakeholders via telephone, Zoom or Teams. Ninety-two interviews were recorded, semi-structured and supported by a topic guide where questions centred on experiences at each stage of the investigation, involvement at each stage of the investigation, and use of the guidance (average interview length 43 min). However, due to the sensitive nature of discussion they were flexible to focus on relevant topics participants chose to discuss. Thirty-five interviews were unstructured, ethnographic-style exploratory discussions and accounted for within fieldnotes, along with details of 44.5 h of observations of relevant activity (e.g., investigator training, meetings where the guidance, investigation processes and/or followed investigations were discussed). A distress protocol supported participants and researchers due to the sensitive nature and proximity to the harm incident being investigated (30).

### 2.3 Sampling

#### 2.3.1 Sites

Sites were selected to reflect variability in size, speciality, and patient socioeconomic profile, as well as being guided pragmatically based on locality to researchers. Sites varied, but the independent investigator body (IND) was most distinct (see Table 1). Hospital Trusts were organisational units within NHS England serving a local geographical area providing acute physical (PH1, PH2) or mental health care (MH1, MH2). The national body investigated maternity-specific incidents that met certain criteria of harm as part of a national strategy to improve maternity safety. Families consented to their details being passed to the independent body by the NHS hospital Trust where the incident happened. Independent investigations tended to be assigned to a lead and a support investigator from a regional team based on caseload. Investigations follow a structured process which involved several panel meetings to review the investigation and report. Investigators were supported by centralised support and resources. Investigation processes at Trusts varied, but there were similarities. On declaring an incident, processes across Trusts centred on reviewing cases logged on web-based software (Datix) and discussing with clinical, patient safety and/or governance staff. Some cases were also raised by the coroner. Some Trusts held meetings dedicated to discussing specific incidents e.g., those resulting in moderate-severe harm or death or those being investigated. The way that investigators were assigned at Trusts varied based on the model used locally. Some had dedicated teams of full-time investigators, others supplemented clinical staff roles, or relied on bank staff working on a temporary basis. Investigators self-selected or were assigned based on their clinical expertise and/or capacity. Support and resources at Trusts varied but was generally limited.

**Table 1:** Contextual site summaries.

	PH1	PH2	MH1	MH2	IND
Organization summary					
**Setting**	Physical		Mental health		Maternity—National
**No. of patients**	Approx. 500,000	Approx. 800,000	Approx. 580,000	Approx. 980,000	Approx. 700 consenting families meeting criteria of harm per year
**No. of sites**	6	8	2 (largely community care)	50	307 (all maternity units in England)
**Socio-economic profile**	One of the most deprived areas of England	One of the most affluent areas of England	One of the most deprived areas of England	A mixture of affluent and deprived areas	National reach across England
Investigation process					
**Investigators we worked with**	Investigators had varying levels of experience, training and caseloads. Incidents deemed complex tended to be investigated by those more experienced. Investigators also worked in governance or clinically. Some maternity staff conducted regular investigations	Predominantly one bank investigator, whose role was purely to investigate and who conducted most Trust investigations. They were trained, experienced in patient safety and had a background in nursing. Other investigators had current senior clinical roles with limited time to investigate	Investigators were employed at the Trust and bank investigators who were used regularly and investigating was their only role. Investigators had varied backgrounds in patient safety, complaints, audit and nursing. Levels of training varied. Investigations tended to be assigned based on caseload	Investigators were employed at the Trust and bank investigators who were used regularly and investigating was their only role. Bank investigators included retired members of staff who investigated on a part time basis. Levels of training varied. Investigators had varied backgrounds in patient safety, national investigations and nursing	Independent investigators worked in pairs, and their role was purely to investigate. Investigators were supported with central resource and training. Investigations were assigned based on region and caseload. Investigators had varied background in midwifery, nursing, academia, safety science, governance, patient safety, risk management and inclusion
**How patients/families were involved**	Investigators made initial contact to explain the investigation process and ask how they would like to be involved, usually via telephone. The investigator provided updates as regularly as agreed		Investigators liaised with clinical teams to determine if any contact had been made (e.g., condolences) before explaining the investigation process, usually via letter, including contact details if they wish to be involved. Where necessary, the investigator sought next-of-kin contact information via the coroner. The investigator asked those who made contact with them, how they would like to be involved	The care team made initial contact and introduced the investigator where possible. Investigators explained the process and asked how they would like to be involved via a method that felt appropriate e.g., letter, telephone or meeting. Where necessary, the investigator sought next-of-kin contact information via the coroner. People were invited to contribute to setting the terms of reference and the investigator provided updates as regularly as agreed	The investigator and investigation support made initial contact, usually via telephone, to arrange an in-person meeting in the family home where possible, to listen to the family perspective of what happened and to explain the investigation process. The investigator provided updates as regularly as agreed, guided by a 10-step process
**Sharing the draft report process**	Not formally part of the investigation process—tried once and was signed off by the executive team before sharing with the family. The executive team routinely gave final sign-off of reports	The draft report was accuracy checked by a clinical team and permission was sought from a subset of the serious incident group to repeat the process with the patients/family if they wished to receive it. Feedback was considered and changes made where necessary. Investigators present the report to the serious incident group for final sign off. A copy is sent to the patients/family by post. Liaison is then handed back to the care group	The draft report was shared with the clinical team. Patients/relatives were notified that the report was ready to share and asked if they would like to receive a copy, usually via letter. The final report was provided to the coroners where necessary	The draft report was signed off by the quality assurance group and shared with the patients/relatives if they wished to receive it, via a method that felt appropriate. The Trust incident review group provided final sign off of the report and sent to the coroners where necessary	The draft report was reviewed by a panel of clinical advisors and sent to the Trust for accuracy-checking and any amendments were made, before repeating the process with the family. The final report was shared with the family, Trust, NHS Resolution and relevant organisations. A tripartite meeting was offered. If accepted, the Trust was responsible for arranging representation from the Trust, independent investigatory body and family

#### 2.3.2 Investigations

Sites notified researchers of any newly initiated investigations. Together with the lead investigator, it was decided if the investigation was relevant and appropriate to follow. This was an iterative process of leaning on the investigators’ expertise, considering the practicalities and sensitivities of the case, training the investigator where necessary, and using “information-orientated selection” (31) to gain variation on variables including setting, service, assigned investigator, and level of harm. This approach was designed to “maximize the utility of information from small samples [with] cases selected on the basis of expectations about their information content” (31). Of the 74 investigations discussed, 29 were followed (see Table 2). Investigations related to suicide (9), therapeutic cooling at birth (4), neonatal death/stillbirth (2), retained surgical item (3), unexpected death (2), attempted suicide (1), neonatal cardiac arrest (1), displaced pacemaker wire (1), death following fall (1), wrong-patient procedure (1), missed maternal tear (1), missed diagnosis (1), infection at cannula site (1), and self-harm (1). Reasons for not following investigations included: investigations being assigned to an untrained investigator who did not consent to being trained, investigations having limited potential for patient/family involvement or ongoing police involvement, delays meaning the investigation fell outside of the data collection period, collaboratively feeling that the sensitivities of the case made it inappropriate to follow and wanting to achieve more variation in the sample. For 20 followed investigations, only the investigator was interviewed. For 8, the investigator and patient/family were interviewed and for 1 the investigator, patient/family and staff were interviewed.

**Table 2:** Recruitment summary.

	PH1	PH2	MH1	MH2	IND	Total
**No. of trained investigators**	9	7	10	6	17	49
**No. of investigations discussed**	22	10	8	18	16	74
**No. of investigations followed**	7	4	2	10	6	29
**No. of investigators we worked with**	3	1	2	3	7	16
**No. of people consenting to take part**	14	11	5	11	20	61
**No. of patients/families consenting**	3	2	0	2	5	12
**No. of staff (non-clinical, clinical) consenting**	4 (2, 2)	3 (3, 0)	2 (2, 0)	2 (2, 0)	2 (2, 0)	13 (11, 2)
**No. of interviews**	38	23	7	19	40	127

#### 2.3.3 Investigators

49 investigators were trained in using the Learn Together guidance. Sixteen of those provided the guidance to relevant patients, families, or staff and invited them to take part in the research where appropriate, in at least one investigation they led on that we followed. These 16 were interviewed longitudinally.

#### 2.3.4 Patients and families

Given the practical and emotional challenges experienced following healthcare harm, an “open-door” consent approach allowed people to join the study at a time that felt right, change their mind about taking part or not take part in the study. Twelve people (2 patients, 10 relatives) were recruited, relating to nine investigations. Relatives were parents, children, grandchildren, siblings, spouses, or cousins. Given the focus of the study, exploring cases of non-engagement were important, and so data relating to investigations where patients/families were not recruited were included.

#### 2.3.5 Staff

Clinical staff involved in the incidents being investigated were recruited in the same way as patients and families described. Investigators approached and provided 12 clinical staff with the guidance and two consented to take part in an interview. Relevant non-clinical staff were identified via chain referential sampling. Eleven non-clinical staff were approached and nine consented to taking part in eleven interviews relating to organisational processes and contextual factors, rather than a specific investigation.

### 2.4 Investigator training, setup activities and overcoming issues

Forty-nine investigators were trained by the research team (17 at IND, 10 at MH1, 6 at MH2, 9 at PH1, 7 at PH2) via a 2-h virtual session to introduce the guidance and underpinning evidence and provide opportunity to discuss how it might work locally, as well as develop a community of practice. The study was presented to senior leadership at three sites (PH1, MH1 IND) and NHS England provided a letter of endorsement to Trusts and relevant governing bodies. All sites were given a welcome pack including copies of the guidance, branded stationary, a site-specific flow-chart, and recruitment material. Throughout fieldwork, a regular email was sent to sites to update on recruitment and discuss emergent issues, resulting in two additional meetings with sites struggling to recruit (MH1, IND). Additionally, nine workshops (see Table 3) discussing and deepening our understanding of emerging issues and developing solutions to overcome them were held. Topics were determined based on issues identified by the research team and participants.

**Table 3:** Workshops throughout the evaluation.

	Stage of fieldwork	Format	Attendees	Workshop topic
**Workshop 1**	Early	Virtual	Trained investigators across sites and research team	Sharing a draft investigation report with patients and families
**Workshop 2 and 3 *Same content delivered twice***	Mid-way		Trained investigators and managers across sites and research team	Recruitment, sharing a draft investigation report with patients and families, adopting a more joined-up approach, and additional complexities in mental health settings
**Workshop 4**	Late	In person	Trained investigators and managers across sites, patients/relatives, co-design partners, academics, policymakers and research team	Revising the co-designed Learn Together guidance
**Workshop 5**				Sharing a draft investigation report with patients and families
**Workshop 6**				Involving patients and families in a mental health setting
**Workshop 7**			Trained and un-trained investigators and managers working for the national investigation body and researchers (LR, JOH).	Involving families in independent investigations
**Workshop 8**			Researchers (LR, JOH, JM, DH)	Revisions to the guidance and development of supplementary website, imagery and video content.
**Workshop 9**			Researchers (LR, JOH, JM, DH)	Revisions to the guidance and development of supplementary website, imagery and video content

### 2.5 Analysis

Interview and fieldnote data were transcribed. Two authors (LR, DH) led the qualitative analysis, with weekly discussion between researchers (JOH, JL, SMcH, RSE, JM) and monthly support from expert qualitative researchers (LS, JW) prior, during and after data collection to discuss initial impressions and deepen understandings. An adapted version of pen portrait methodology (32) was used to collate complementary data sources for the purpose of completeness, and to support analysis. Pen portraits are a technique to integrate multiple sources, and large volumes, of qualitative data into a concentrated account, focussing on a given topic. Data were first organised according to each investigation, nested within a wider contextual case report relating to each site which was collated within a working document. Notes of initial impressions were made, and researchers explored the similarities and differences within and between sites via open and thematic coding. This helped to develop descriptive accounts of the common and distinct processes of investigation and the expectations and experiences of stakeholders in these processes. Interesting foci, both specific to the research questions and those capturing novel ideas, were integrated to form the basis of a pen portrait, which was iterated until a consensus was reached. The representation of data sources was not necessarily equal, and all sources were not necessarily represented, but included dependent on data quality and significance to the foci identified. A multi-case thematic analysis of the pen portraits and contextual case reports was then conducted, adopting a reflexive approach (33). This approach recognised the importance of considering linked typical and atypical cases and drawing higher-level conclusions to understand complex phenomena (34). Analysis was structured according to the foci identified within the pen portraits. At each stage of analysis, decisions were discussed until a consensus was reached, data were revisited, and conflicting accounts were taken account of.

## 3 Findings

Overall, investigators and organisations were supportive of the guidance, agreeing that meaningfully involving patients and families felt like the moral thing to do. An important external enabler was the timely policy transition within (35) from the Serious Incident Framework (SIF), to PSIRF, with an emphasis on compassionate engagement. This led to relevant internal and national conversations, as well as various re-structuring efforts and adaptations of processes. Despite similarities, we found that some of the underlying constraints surrounding involving patients and families were different, or more pronounced; between settings, when different staffing models were adopted, depending on the nature of the incident being investigated, the resource and capacity of the teams, and also the various disciplinary backgrounds, levels of experience and capacity of individual investigators. Sites also had different histories of involving patients and families in investigations, which influenced current attitudes and beliefs. Some of the nuance associated with these factors is explored according to three key stages of the investigation below; (1) Inviting engagement and involvement (2) Gathering information and (3) Sharing the report.

### 3.1 Inviting engagement and involvement

The guidance supported investigators to engage with patients and families, sensitively inviting them to become involved early in the investigation, where possible. However, complexities were apparent across settings, perhaps exacerbated in mental healthcare, partly due to the nature of incidents being investigated.

“We predominantly have deaths as our main serious incidents, just due to the nature of kind of, you know, mental health Trusts” Patient Safety Manager, MH1.

Within hospital Trusts, investigators often had to conduct “pre-investigations” to source the appropriate details of people to contact, juggle ethical dilemmas of involving vs. re-traumatizing, and work within contexts of unclear organizational processes and responsibilities. These issues were largely circumvented when investigations were conducted by an independent body, due to better established processes, infrastructure and resources. These are explored in detail.

#### 3.1.1 “Pre-investigation”: who and how to contact

The guidance supported the systematic involvement of patients and families in investigations and informed them how, why, and when to be involved. However, this was not always simple.

“We've still got a little bit of learning to do in terms of making sure that we're consistently sending out the information at the right time… that often is because initially we don't have the contact details and then sometimes it can kind of slip off the radar a little bit… we've still got a little bit of work to do around that.” (Patient Safety Manager, MH1)

Knowing who and how to contact the relevant person or people sometimes felt like an informal investigation in and of itself, made particularly difficult with limited capacity (e.g., investigating in addition to a clinical role), experience and training. Cases of death also often required liaison with different services, care teams and the coroner's office. Piecing together potentially incomplete, outdated and/or conflicting information was a challenge. Some staff described a sense of obligation to protect patients which was sometimes tied up in confidentiality tensions, multifaceted family dynamics and fractious relationships. Other issues included missing, outdated or conflicting next-of-kin information on NHS systems, elderly or otherwise vulnerable next-of-kin and multiple people wanting to be the main point of contact on behalf of the family.

“You have to tread carefully because you don't know what is going on in people's lives. Divorce, estrangement, how much does the person who has died want them involved? We feel a responsibility for the person who has died. We're protective of them. It can be an ethical minefield. We sometimes have different next-of-kin information to the coroner—what do we do then? The coroners’ office is overwhelmed with backlog and responses aren't always quick. Family dynamics are quite often difficult and relationships are strained.” (Patient Safety Manager—verbatim fieldnotes, MH2)

Some explored formal and informal routes of cross-referencing information, but felt uncomfortable making forced, rushed and difficult decisions based on limited knowledge. Because of this, some investigations progressed without inviting involvement, or there were significant delays in anticipation of it. This meant that any potential benefits of the guidance were deferred or could not be seen until it was arguably too late.

“I haven't made any contact with the next-of-kin for that reason—it's documented as a friend…she was in hospital for a couple of weeks and she fell at some point which was when she sustained a fractured neck of femur and the next-of-kin, when he was contacted, didn't know that she was even in hospital, so I'm not feeling it's a very close next-of-kin and therefore we've made the decision not to contact him about the investigation.” (Investigator, PH1)

Additional challenges included where the family were reluctant to be involved for reasons of self-blame or shame, or cultural reasons to not recognise or acknowledge the incident.

“A family would be really reluctant for people to find out that a member of their family took their own life during Ramadan. Culturally, this is not socially acceptable and so the family would not want it spoken about in court because this could lead to newspapers finding out. Trying to engage with a family in these circumstances would be very difficult.” (Investigator—verbatim fieldnotes, MH2)

Many of these issues were circumvented in a national investigatory body context, as NHS Trusts provided contact information once families consented to their case being investigated. Typically, where possible, parent(s) were also the obvious stakeholder to be involved in maternity related investigations, in addition to other close relatives where appropriate.

#### 3.1.2 Juggling the ethical dilemma of involving vs. re-traumatising

Investigators sometimes felt responsible for juggling the ethical dilemma of inviting involvement early, but also not wanting to overburden or re-traumatise those who did not want to, or did not feel able to be involved due to the sensitive circumstances, which often needed to be inferred. This sometimes form a significant part of the investigator's role.

“We kind of had partner, mum and sister, who were all really vocal, wanted to be involved, and that's okay, but none of them spoke to each other, didn't have a good relationship… mum was saying don't share it with my daughter, daughter was saying don't share it with my mum, and then we had partner on the other hand, who was a service user and really struggling and she went onto hurt herself after speaking to me. So then mum and sister were saying don't share it with her anymore, she's not okay, she's saying I want it… the mental health side of things…there's some really difficult dynamics to try and manage… when somebody's struggling with their mental health it does fracture relationships… It's not isolated just to mental health incidents, I'm sure. But I do think it is amplified.” (Investigator, MH1)

Attitudes to this dilemma were also informed by historical approaches and experiences of involvement. For example, both mental healthcare Trusts had a history of an “opt-in” approach to involvement. This meant that a letter was sent providing contact information and in cases of no follow-up, it was assumed that families had made an informed choice to not be involved. Instead, in some instances, investigators suggested that it meant that the invitation had not been received by those who did want to be involved, they were unable to comprehend the invitation at a difficult time in their lives, they did not trust that the organisation would listen to their views if they did become involved, or they did not want to be involved temporarily to allow for grieving. The guidance encouraged and supported an open-door, opt-out approach, but those with less experience felt nervous and ill-equipped. One of the mental health Trusts transitioned to this and saw an increase in involvement. The change was also prompted by experiences of families asking to be involved on later receiving the investigation report, often via the coroner or upon request from a legal representative. Families had reported that finding out information for the first time via an external source indicated that the organisation did not care, nor wanted to learn from what had happened.

Within the context of national investigations, positionality differed, coming from the standpoint of independence. While the risk of re-traumatising families was a concern, the relative luxury of gaining the confidence of families was perhaps afforded, in comparison to investigators employed by the Trust where the harm event occurred. With independence, greater resources and time, investigators were instead, sometimes perceived as a “saviour” for the family.

“Investigators often come from healthcare, they have got that very emotional nature and they're compassionate, they're considerate, they walk the mile in those person's shoes. It has a big effect on them and how they want to communicate with the families and that can be beneficial but it can also be very negative for both parties because families can become very reliant on the investigator and the investigator can also become reliant on the families, yeah, because they see themselves as being a saviour.” (Family Engagement, IND)

#### 3.1.3 Unclear organizational processes, roles and responsibilities

The guidance provided overarching principles of involvement, however, organisations were locally required to establish their processes, roles and responsibilities. Where these remained unclear or changed part-way through an investigation, patients and families were left to make sense of the reasons why.

“I've had contact. She did send an email… She was helpful but that is the only time I've actually heard from them… Now, I don't know if that's just a normal thing because I suppose they can't just keep ringing you up or emailing… you don't want to just keep ringing up and asking, do you? I would prefer her to ring me and say this is where we're at now and this is what we've found out so far… we've just had Mother's Day and I know it's a trivial thing but Mum got two cards instead of three… it makes it more painful…the waiting and the not knowing… It can feel a little bit like it's easy to not involve people after any progress, hoping that they'll not make a fuss or question. And I'm sure that's probably not the intention but it does feel like that.” (Relative, MH1)

Issues sites encountered included; unclear responsibilities for stocking and storing the guidance, remote working, leave or a change in roles cause delays and disruption, disagreement about how and when the guidance should be introduced, and lacking clarity about who was “eligible” to receive it. This was experienced negatively by patients and families, and emphasised the need for a joine-up approach, in which all levels of the organisation understood and valued the process, underpinned by adequate resource.

“Following the incident, the patient received a letter from the Trust apologising for what happened, confirming that an investigation was being launched and outlining estimated timelines. However, no other information was provided at this stage. Due to delays assigning the investigation and disrupted processes, the patient was left feeling confused about what was going to happen next. Prompted by the research team a few weeks into the investigation, the assigned investigator sought to send a copy of the guidance. As a bank investigator working remotely, they were unable to determine where the Trust copies of guidance were kept. Instead, they asked a member of the administrative team to print a copy and send it via post, as well as providing a digital copy by email. The patient received the guidance six weeks post-incident. The patient suggested that information would have been useful if it was provided sooner.” (Fieldnotes, PH2)

“So that in-between, for me, is not really good communication. You don't really know what's going on, you don't know what the next steps are, so that for me was like the biggest thing.” (Patient, PH2)

Most operational issues were able to be resolved over time across sites. Within a national context, issues were largely offset by having an existing established, clear and standardised 10-step process, which guided families on what would happen and invited their involvement at key stages. Early in the process, an initial meeting between the family and investigators was held, often face-to-face in the family home. This proved hugely important for setting the tone of the investigation, in which the role of investigators was to actively listen to the family perspective about the issues they chose to raise, allowing them to feel heard. Often, at least some element of what families described during this meeting fell outside of the “terms-of-reference”, but was sometimes the first time following a traumatic event that those experiences had been validated. Giving space for this, whilst not necessarily being able to address the issues raised, appeared to contribute to a dignified, family-centred approach.

“[The family meeting] is not expecting them to walk into the very place where something tragic may have happened… it's putting the families as a credible part of the investigation, not an afterthought, not a, you know, attitude of ‘there, there, there, there, I'll speak to you because I've got to do a duty of candour’… it's actually valuing that they've got a really important part to play… I know that comes with time and resource pressures but I think it's a fundamental part of doing it with any form of meaning really. It's crucial.” (Family Engagement, IND)

Without referring back, families did not necessarily know what each step of the process entailed, but having 10-steps provided a useful shared reference point for families and investigators, to hang ongoing involvement from, as well as providing initial reassurance that the procedure would be thorough. This was something it seemed that Trusts could learn from.

“They sent us something by email… a 10-step thing of like all the main processes that they kind of go through to make the report that will eventually appear… it's felt like quite a robust process… it did seem very comprehensive.” (Relative, IND)

“We all know that families that are distressed don't remember everything they're told or don't look at everything that's written, so to have a one page visual where I can say to you, you know ‘when I spoke to you 2 weeks ago I told you this is what we were doing, on that diagram we're now at step 5, step 5's going to take a couple of weeks and then I'll ring you Monday when I'm hoping we're moving towards step 6, and step 6 is when I can do this’.” (Family Engagement, IND)

### 3.2 Gathering information

The guidance aimed to support investigators, while working to gather information from various sources, in preparation for writing the investigation report. Some experienced investigators who felt familiar with the content used it as an “aide-memoire”, whereas others engaged with the content in greater detail or used it prompt a larger cultural change within the team. Challenges associated with this stage of the investigation included fluctuating involvement and independence affecting relationship dynamics between families and the hospital Trust. These are explored in detail.

#### 3.2.1 Fluctuating involvement

The level of patient and family involvement was rarely consistent as investigations progressed. Sometimes this fluctuation was instigated by patients and families themselves. Some felt that the initial phase of the investigation was “information overload”, and others suggested that they would benefit from more regular prompts to access relevant information at the time it would be useful for them, and to have a supplementary digital platform.

“If there was something to kind of explain it more… a web page… a basic overview of what this process entails… it was still a couple of weeks before I was contacted after that letter so there was still a few weeks where you're in limbo. I do think something like that would have definitely helped.” (Patient, PH2)

Despite intentions of engaging with every detail of the guidance, this was often not possible due to the demands of life at what was often a very difficult time, and the paper-based format meant that it was not always readily available when they needed it, or did not cohere with their usual way of recording information.

“We usually record things kind of digitally, so we might have liked a Word document or something rather than doing it on paper… What I would probably do, is want to fill it all in digitally but then once it was done print it… so that I did have kind of the ease of filling it in digitally, but then I have like an actual physical thing as well.” (Relative, IND)

Some, including those enthusiastically involved initially, openly spoke about the reasons that their involvement may decrease beyond practical reasons, such as preserving their emotional energy, or managing simultaneous processes (e.g., litigation, complaints, or inquests), as well as life demands. Others decided to step back when they felt that the investigation had served its purpose, or no longer met their needs.

“If I feel like I'm flogging a dead horse I'm not going to chase it. Because for me, none of this is about anything other than I have been robbed of my best friend and my wife, and my daughter, whose 4 years-old, has been robbed of her mum…that is what I'm trying to get some form of justice for, it's not for anything else and I will not chase it. If someone says to me, you're not going to be able to achieve anything from this, then I will say, fine… I'm not going to chase something that's not there.” (Relative, MH1)

However, often, investigators were left to make sense of the reasons that involvement may have fluctuated and decide how to proceed based upon those assumptions.

“The family were initially very engaged. They are both nurses and work within the hospital in which they lost their baby. I felt we had a really good rapport the first few times we spoke and we still had the same rapport so I don't, I think, I know that she was, she's waiting for counselling and she feels that the wait has been too long and she could have benefited from it earlier but that's just the waiting list of the Trust so I don't, so I think everything's fine. I think she maybe just disengaged because it was just quite difficult and also nothing was really happening.” (Investigator, IND)

Without knowing the reasoning, some investigators referred to feeling discouraged by unrequited attempts to engage.

“I feel quite disheartened by this particular case, with that lack of involvement when I've tried my hardest to keep her involved.” (Investigator, PH2)

Conversely, fluctuations in involvement were sometimes instigated by investigators or organisational processes. Investigators had to navigate challenges including hearing multiple conflicting perspectives, judging the weighting of perspectives and determining what should be included or omitted from the report. This was particularly difficult when it was deemed impossible to reconcile a coherent narrative of what happened and workloads were high, sometimes leading to reduced engagement. However, the guidance helped to support investigators in accepting subjectivity and making clear the nuance.

“I haven't contacted them yet because if I'm honest, I've been putting it off because we've had a clinical lead get involved and he absolutely feels that categorically, you know, this dressing has not been retained during surgery, but I know that the lady's husband's view is that it has… I'm avoiding that difficult conversation to be honest. I'm thinking about inviting them in but getting the consultant anaesthetist that's investigating with me to meet with them together and then we can feedback what we know and we can go from there really.” (Investigator, PH1)

Some patients and families also felt left in the dark when investigators were on leave or changed roles, staff turnover was high, and investigator caseloads were moved between the team. Despite a team-based training approach, these issues appeared to undermine what the guidance was aiming to achieve.

“A pack came in the post which was sent from [the first investigator assigned], actually, and that was just really so that you could write things down as the investigation progressed. But when you have no contact, there's nothing to write down.” (Relative, MH1)

Within the context of independent investigations, initially setting the tone relationally with families was experienced positively. However, expectations were sometimes then heightened. While investigators were gathering information, families were not necessarily involved. For some, updates about the process were enough, however, one family described this jarring switch from a relational focus, to a procedural focus, as “procedural breadcrumbs”.

“The reason why I developed [the 10-step process] was because the beginning of the process can be really busy for families, the end of the process can be really busy, but in the middle it can look as though they're forgotten. They're not, but in the middle the report writing, the quality assurance, the clinical panels are all happening.” (Family Engagement, IND)

From an investigator perspective, this period was also challenging when families were asking for more than they felt ready or able to share with confidence, whilst also feeling an ethical obligation to not unnecessarily withhold information.

“Until you've finished your investigation and until you've been through all your panels and it's been agreed that yeah, this is what we're saying, you don't want to give anything out to a family too soon that might change as the panels go on…but it's how much do you tell them because you don't want any surprises at the end, you don't want them to wait longer than they need to.” (Investigator, IND)

#### 3.2.2 Gaps created by independence

Unlike in Trust investigations, where once an incident was declared, relationships aimed to be built between hospital staff and patients and families, in an independent investigation, this process was disrupted and there were multiple points of contact. The absence of formal policy to support navigating this disruption sometimes created uncertainty for everyone involved.

“I don't know if the input from the Trust is going to ultimately long-term help or hinder me, I don't know, I don't know if we'll say the same thing or if we're going to say different things.” (Investigator, IND)

In part, this was because it was not uncommon for families to continue engaging with the hospital for ongoing treatment, perhaps coming into contact with staff who cared for them under formal and informal circumstances. While independent investigators could control how they worked with families within an investigation to some extent, this was only part of what families experienced. A sense of what was right and wrong in these circumstances was not necessarily clear cut, and risked placing investigators, healthcare staff and families in uncomfortable, and ethically compromised situations, without the appropriate support.

“There is no legislation…what is there to stop a Trust looking into it, in their own way, to improve things?…In a way it's right for them to do it, it's right for them to gather information and do some learning… Trusts might have identified a lot of these things themselves and put those remedial actions in, all to the benefit of patients…. So should they just wait for [the independent investigatory body]? No, I don't think they should and I think that it would be wrong for them to do so but whether that's a personal opinion or whether it's the right opinion, I don't know.” (Family Engagement, IND)

### 3.3 Sharing the report

The guidance encouraged investigators to share a draft copy of the investigation report with patients and families, prior to finalising, to gather their feedback. Neither acute Trust had prior experience of this, however both mental health Trusts had done this sporadically, and the independent body shared draft reports with families routinely. The act of sharing the draft report was found to be perhaps symbolic of something more fundamental and was made challenging for investigators who felt that they navigated systemic barriers alone. Organisational learning was also a challenge. These are explored in detail.

#### 3.3.1 Draft report: symbolic of something more fundamental

The act of sharing a draft report arguably signified the underlying organisational values and ethos surrounding patient and family involvement. Generally, investigators and their teams felt that it was a morally good thing to do, underpinned by a formal expectation set out in national policy—PSIRF. However, concerns and procedural issues meant that this stage was often the thing “to give” when investigators felt under pressure and faced time and resource constraints. Some described feeling forced to offer a compromised version of involvement. This was perceived to be unfair for some families, illuminating conflict between the guidance and organisational pressures to be timely and conduct investigations with limited capacity.

“It was the first time we'd sent the family a copy of the draft before approval and I had asked their feedback. Now they hadn't had particularly long to feedback, they just had a week, ideally two weeks, but time only allowed one week.” (Investigator, PH2)

“We felt, because it took such a long time for the report to be done, that for us only to then be given a week to respond to it, with a bank holiday included, it didn't seem very fair… It kind of felt pressurised because I know that [our investigator] has to then have the final report done by [date]. So it feels a bit late for us to now ask questions, for her to go back and find the answers to those, when it's all got to be completed within seven days now. I suppose there isn't the opportunity for those questions to be answered.” (Patient, PH1)

This was despite having the potential to restore faith in the organisation, or shatter the trusting relationship that may have been built with the investigator. Some explicitly described that their decision to litigate was based on how the organisation wrote their report and dealt with any subsequent questions, suggesting that investing in this stage of the process may avoid the workload needing to be absorbed elsewhere in the system (e.g., formal complaints, legal teams).

“The granddaughter of the patient who died had collated questions, together with her mum and wider family to send to the investigator. She suggested that she would wait and see how they were responded to, to inform what she would do next—nothing, or take legal action.” (Fieldnotes, PH1).

There was also disagreement surrounding what constituted a “draft” report, requiring different levels of sign-off within and between organisations, before it was deemed acceptable to share. Some struggled with balancing the concerns of sharing a draft report subject to significant change too soon, and sharing a “draft” only once it had been given final organisational sign off.

“I think the idea of sharing those draft reports, to get the comments, is good. I think it's also done in the right way by giving it to the Trust first and again, that can be controversial but I think it's right because when you present it to the family, you want to have it as complete as possible with the least errors as possible, otherwise all you're doing is changing and changing and changing again. That undermines confidence in the organisation, it undermines confidence in the investigators and the actual family are being put through more stress than they need to.” (Family Engagement, IND)

#### 3.3.2 Navigating systemic barriers alone

Challenges with sharing a draft report with patients and families were largely absorbed by individual investigators. This was on top of the emotionally laborious role of acting as a “buffer” between distressed patients and families and organisational pressures, and the upfront workload required to prepare for, discuss, and deal with any resulting feedback and questions. While the guidance was able to provide overarching principles and procedural support at this stage, it required organisations to consider reorienting their infrastructure and ensure that their culture was receptive to change. Without that, investigators carried the burden of having to win the hearts and minds of management and the wider team that this was something that was of value, and were left to navigate systemic barriers alone. This could feel like a “thankless task” when patients and families were left unsatisfied.

“I spent a lot of time I suppose, taking the draft report to a committee, for that committee to then have to read it to give me permission to send it out. Other people then having to password protect it, then emailing it out and then hearing nothing… It hasn't changed the content of the investigation, but it's created more work for me and others.” (Investigator, PH2)

In other cases, sharing the draft report led to unintended consequences. For example, by giving the family more time to feedback on the draft report in one investigation, the report was not made available to the coroner, meaning witnesses could not be called to coroner's court. Other times, investigators struggled to manage the feedback received, particularly when it fell outside of the terms of reference. To make this stage of the process more easily manageable, some were keen to boundary the elements that patients and families could feedback on. At the independent investigatory body, this process was referred to as a “factual accuracy check”, in which families were invited to reflect on their account, but not provide feedback on the clinical findings. Views varied about how effective this approach was, with some having previous experience of families presenting legitimate clinical challenge, leading to changes in the report. It was clear that more support surrounding the sharing of the draft report for all stakeholders would be useful.

“It's not factual accuracy, actually, usually they challenge opinion or our analysis, which we say is not challengeable, but sometimes it is. Sometimes we get the wrong information from our clinical advisors and we have to change our analysis, that's happened… but that's just the fallibility of, you know, knowledge, I suppose, isn't it…It's just another opinion, after all. Our advisors are current, they're in practice and they're high up in their game, but there's nothing to say that their word is absolutely final… There are some experts, I suppose, that people rely on, but sometimes it is about opinion.” (Investigator, IND)

Generally, the issuing of the final report was considered the end of the investigations’ team responsibilities and marked the point at which patient and family engagement tended to draw to a close. Where the investigation felt meaningful for patients and families, the guidance was enough. However, where involvement felt tokenistic, some patients and families felt that this was just the beginning for them. This was particularly true for those who felt like their needs had not been met nor their questions answered and pursued alternative routes to meeting those needs such as raising formal complaints or pursuing litigation. Others were also involved in separate but related processes such as coroners’ inquests, resulting in further delays and complication.

“I did think that there would be an inquest but then it sort of, it just prolongs everything, doesn't it? And it's the waiting. I accept that it's not easy to glean all the information… but it is awful, just being in limbo… I feel that [my sister], perhaps will not have justice, as it won't change things… we're all sort of on tenterhooks waiting as to when it might be and it could be ages yet.” (Relative, MH1).

The guidance usefully signposted patients and families to potential sources of support, but was designed to supplement, rather than replace, any existing support provided. Nonetheless, confusion was evident about whose responsibility this was. In a national context, these issues were present, but to a lesser extent as a longer history of sharing draft reports with families had led to a better established, and standardised process in which families were formally allocated time to read and feedback on the report, despite timings being relatively short and feedback often being limited to their account of events. National independent investigators were also solely employed to investigate with the support of a central family engagement team, as well as clinical advice and a regional and national support network of colleagues. Without investigators working within a system which supported them to do this properly. There were risks of compounded harm for everyone, including patients and families, but also investigators.

#### 3.3.3 Organisational learning

The guidance encouraged investigators to set flexible terms of reference collaboratively with patients and families, as well as clear expectations of what the investigation could achieve. Nonetheless, organisational learning as a result of healthcare harm was a challenge across sites, particularly following complex investigations, such as those spanning long histories, involving multiple care providers and external agents or resulting in death. Adding further complexity were staff being unable to inform the investigation due to moving on from their role, or working in agency roles.

“There might be a bit of a delay actually, which I ought to let [the family] know about, because one of the staff members is an agency midwife so she's not been contactable through the Trust.” (Investigator, IND)

Often, patients and families were raising more fundamental issues with care than an individual investigation could address. The impact of investigation reports also remained largely elusive, not only for patients and families, but for investigators too. To help to overcome these issues, efforts were being made to develop closer working relationships with senior representation from the concerned care teams. For example, both mental health trusts had begun regular meetings with clinical managers to discuss the proposed recommendations and advise if they felt reasonable and feasible in practice. The aims were to ensure that the appropriate investigations were being conducted, and that the recommendations were actionable, accounted for local context and could be better communicated.

“Because of the nature of the death under the current framework, it would require us to do a review even though we feel that there's probably not much of an opportunity for learning… for some cases we spend time doing an investigation that doesn't really deliver anything other than saying everything was okay. Now, we're linking that learning and improvement kind of mind-set so that we don't keep repeating the same investigations time and time again, so that we can tell families this is the improvement we're doing, this is where we're at.” (Patient Safety Manager, MH1)

In the context of the investigatory body, a strength of independence was not being constrained by context. However, the nature of being at arm's length made it more difficult to ensure that recommendations were realistic, meaningful and could contribute to organisational learning. This may create further problems for the rebuilding of the trust between families and services, if families feel that services cannot, or will not make changes to reduce the likelihood of what happened to them, happening to someone else. It may also set unattainable goals for staff, which risks disenfranchising Trusts.

“How can we learn effectively unless people have got that, (1) ability to take part in it, (2) confidence to take part in it and (3) knowing that if they don't take part in it we're missing a piece of that jigsaw and that we're reliant on that and therefore they're going to get a slap on the wrist somehow.” (Family Engagement, IND)

## 4 Discussion

This study aimed to undertake a longitudinal, “real-time” evaluation of the use of the Learn Together guidance in practice. To our knowledge, this represents the first study of its kind, i.e., the first attempt to implement and evaluate guidance to support the meaningful involvement of patients and families and gather stakeholders’ views across multiple care settings in the UK. This makes a valuable contribution to the literature, as well as methodologically highlighting the importance of evaluating and refining co-designed materials in practice. As our findings show, even when stakeholders at different levels of an organization are morally signed up to an idea (27, 28), the reality of achieving it is rather more complex. This illuminates that when embedding processes for involving and engaging patients and families in incident investigations and responses, organizations need to first seek to understand how this is currently done and seek to adapt current organizational infrastructure to support them. We were also able to identify discrepancies between what harmed patients and families told us that they would have wanted in hindsight, and what people actually wanted in the moment when they were distressed, grieving, receiving ongoing treatment, had new babies to care for and/or were generally leading busy lives. We were able to reflect this complexity within the final versions of the guidance, both enhancing future uptake and reducing the likelihood of compounded harm for patients and families (10) (see Table 4; Box 2).

**Table 4:** Revisions to the learn together guidance following evaluation.

Revisions to the learn together guidance following evaluation	Explanation
**A shift in *tone* from “information sharing” to “*rebuilding trust and relationships*”**	The original tone of the guidance centered on sharing information to patients and families, to equip them with relevant knowledge and empower them to become involved in ways that met their needs. However, the needs of patients and families varied widely, and frequently changed as investigations progressed. Because of this, the “identity” of the guidance was important, and needed to shift more towards rebuilding trust and relationships. This was driven by the principle that harmed patients and families want human responses to harm, and not organizational responses. This affected how the guidance as a whole was written and led to the addition of an opening letter from members of a patient and family advisory group, compassionately relating to how people may be feeling. In addition, personal messages from people with lived experience were scattered throughout the revised guidance
**A *re-structure* to *orient around relational touchpoints* rather than chronology**	The guidance was originally developed following the chronology of an investigation. This assumed that time would be the most common reference point. However, we found that patients and families were not always ready to engage in an investigation process whether due to emotional state, or practical constraints of everyday life, and that “time” itself can be experienced in a fundamentally different way for people, especially those who recently experienced trauma. Therefore, we shifted the focus from chronology, to relational touchpoints, learning from the independent investigatory body and their 10-step process. This accepted that the “start” point for patients or families might happen after an investigation has started and that their level of engagement may vary at different stages of the investigation. The emphasis was placed on being as involved as much or as little as the patient or family would like, and that regardless of when they wanted to engage in the process, the same relational “touch points” would apply. This understanding sits at the heart of the revised guidance which is guided by a new process—termed the “five-stage process”: **Stage 1: Understanding you and your needs**—The investigator is encouraged to meet with the patient and family to understand their needs following the incident. Patients and families need time and space to talk about what has happened, in their own words. This is an important step on the road to recovery and helps them begin to make sense of what happened. **Stage 2: Agreeing how you work together—**The investigator is encouraged to work with the patient and family to develop a shared understanding of how the investigation will progress, what the investigation will look at and how they will work together based on ten common principles (27, 28). **Stage 3: Giving and getting information—**The investigator is encouraged to gather information from relevant sources, including the patient and family, who have a unique and valuable perspective on what happened and may have information others do not have access to. This step includes regular updates, even if there is no specific news, and being transparent about any delays. **Stage 4: Checking and finalizing the report—**The investigator is encouraged to discuss key findings with the patient and family before passing on the draft report, sensitively preparing them for any information that might be unexpected or any points of disagreement. This is because reading the draft report for the first time can be very difficult, even after such discussions, and the patient and family should be prepared for this. The investigator is encouraged to invite the patient and family to feedback on the draft about the accuracy of their account of the incident and other important details, raise any additional questions or challenge other content. **Stage 5: Next steps—**The investigator is encouraged to close the investigation in a way that dignifies and reflects the potential impact on those involved, provide a copy of the final report and check whether they have any further questions or needs for support. If the investigator is unable to meet the patient and family's needs, they are encouraged to advise them where to access the appropriate support
**Additional *support and resources* for all when sharing *draft investigation reports***	The sharing of a draft report was found to be a critical window of opportunity for enacting the fundamental principles of meaningful involvement and was one that shaped as much by the flexibility of the wider system, as the approach taken by individual investigators. The revised guidance provided additional, detailed support and resources for investigators, patients and families during this stage. For patients and families this included a message from someone who had experienced an incident investigation, as well as sections on “getting prepared for the report” and “checking the report”. For investigators this included sections on “preparing to share the draft report” and “getting feedback on the draft report”
**Explicit reference to the required *support and training* for investigators working in a challenging and *emotionally laborious* role from all levels of the system**	It was clear that there needed to be an explicit recognition of the loneliness felt by individual investigators faced with an emotionally demanding role, as well as navigating systemic issues alone. Because of this, the revised guidance emphasized the importance of not only the investigator, but also middle management, senior leadership and the wider system and outside agents who all play a significant role in enabling or making it difficult for these people to do what feels like the “right thing”, and meaningfully involve patients and families following patient safety incidents
**Supplementary, digital resources**	A new, supplementary website containing videos and images from lived and professional experience, as well as additional signposting to relevant support for all stakeholders was developed (https://learn-together.org.uk)

The findings support previous research in suggesting that patients and their families can share credible information regarding the safety of their care [e.g., (4–7)]. However, what came across overwhelmingly was the need for harmed patients and families to heal. This echoes calls made by the Parliamentary and Health Service Ombudsman in a recent report (12) for accountability for a robust and compassionate response to harm, which supports learning for systems and healing for families. It also aligns with recent work in NHS Scotland, suggesting that when meaningful involvement is done well, it can help with reconciliation following a traumatic event and help restore their faith in the healthcare system (36). The guidance was able to go some way to meeting those needs, however, we recognize that further systemic changes are needed to truly support patients and families to heal. There remains a paucity of research to suggest that moving towards restorative approaches to harm, such as those being taken in New Zealand (37) are feasible within the current English healthcare system.

This would involve a much more substantial shift as a healthcare service. At an organizational level, this approach may require both subtle, and larger scale changes to infrastructure, and the wider context they exist within. Our findings clearly indicate that investigators would benefit from further support, and investigation specific training. Equally, culturally entrenched attitudes beyond the individual investigators, or investigations team, need to be aligned and in support of this work, in order for it to stand up against other organizational priorities when falling under time and resource constraints. For example, these activities must be formally recognized as skilled “work”, and people cannot simply do this on the side of a demanding role because they are clinical—at least not well, or in a way that does not compound harm for people (10). In addition, perhaps secondary, tailored guidance focusing on the “pre-investigation” to determine *who* should be involved, and circumstances in which confidentiality could or should be breached would be beneficial, with supportive systems and protected time and space within job plans.

Investigators work as “boundary spanners”, where a significant part of their job involves directly interacting with people and addressing their complex and variable needs under conditions of uncertainty and with potential conflict (38). In other industries, boundary spanning has been found to result in role conflict, role ambiguity and a range of outcomes such as low job satisfaction and intention to leave as well as physical and psychological health issues (39). However, perceived organizational support—that is, “the extent to which employees perceive that their contributions are valued by their organization and that the firm cares about their well-being”, has been recognized as an important factor to mitigate these factors (39). Kalman (38) further suggested that boundary spanners are most likely to be successful when they prove influential within the organization, amongst other factors. Therefore, organizational efforts must ensure that people working in investigator roles are adequately supported and their efforts contribute to making a difference. Without these systemic changes, we may be trying to fit an approach that centers “*people*”, into organizations which center “*processes*”. This may result in patient and family involvement becoming the thing that is the first to go when organizations and individual investigators come under pressure, indicating that it is not *a priori*ty. It is perhaps too early to tell from our data, if the ongoing rollout of PSIRF poses an opportunity, or further challenge, in the meaningful involvement of patients and families following patient safety incidents.

What is clear is that there is no one perfect organizational model to meet the needs of patients and families. Rather, each comes with a set of benefits and challenges which must be balanced and considered in light of local organizational context. Instead of the role of individuals fulfilling the duty of patient and family engagement, these decisions should be grounded in the ability to meet their needs, as well as the needs of other key stakeholders. Regardless of the organizational model opted for, the responsibility of patient and family engagement should also be recognized as part of a professional and skilled role, with protected time and space, underpinned by relevant training and support. What our evidence was unable to evaluate, was the increasingly established Patient and Family Liaison Officer role, which require further research to evaluate its effectiveness. An evaluation of the FLO role did however, find that lacking organizational support was a significant issue (40).

### 4.1 Revised learn together guidance

Based on the ethnographic evidence, the guidance was significantly revised in a number of ways. These revisions largely centered on five key requirements of the guidance (see Table 4; Box 2).

Box 2 **Revised learn together guidance and five-stage process (27, 28). Reproduced with permission from “Learn Together Resources” by Jane O'Hara, licensed under CC BY 4.0.**

*For patients and families:* 
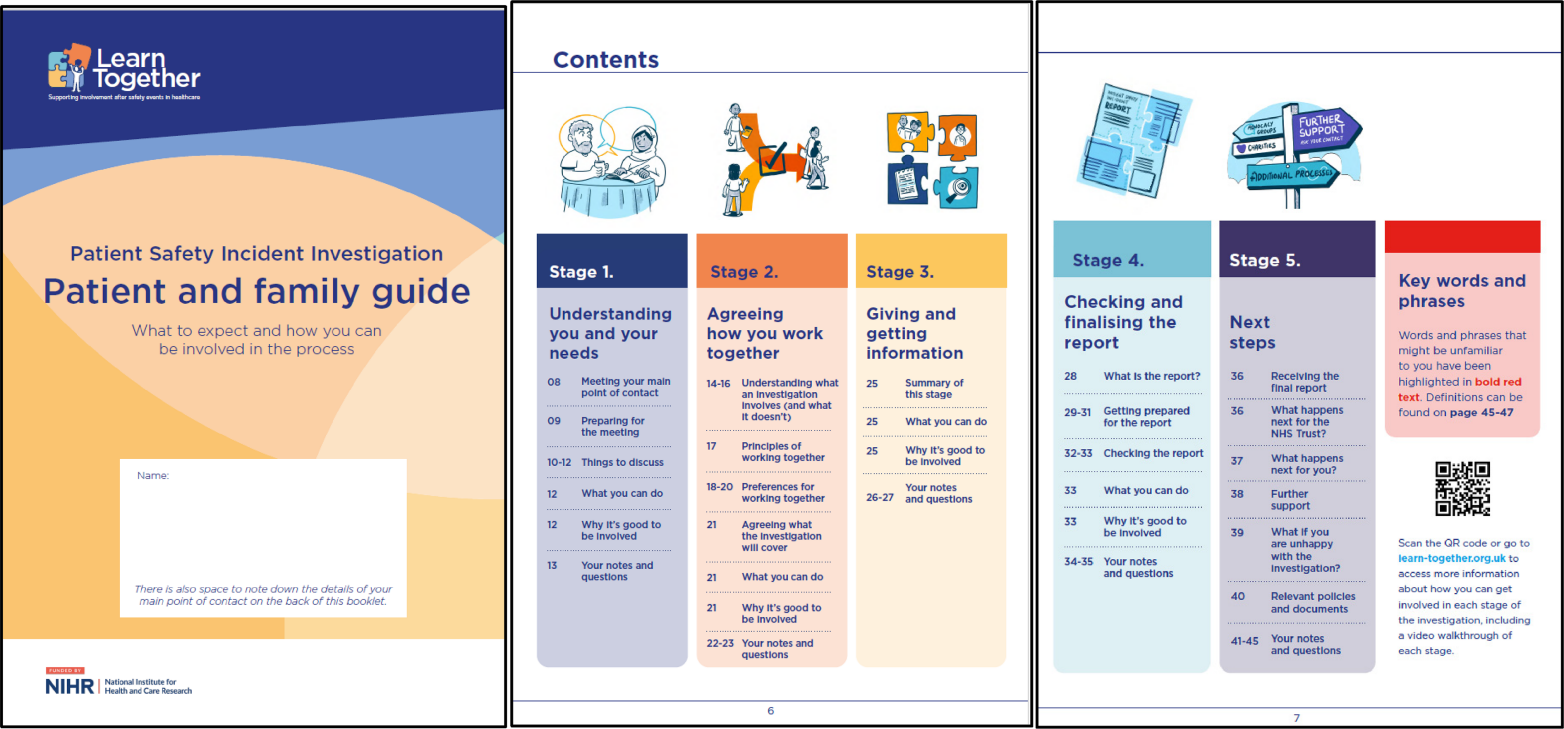
 *For investigators and engagement leads:* 
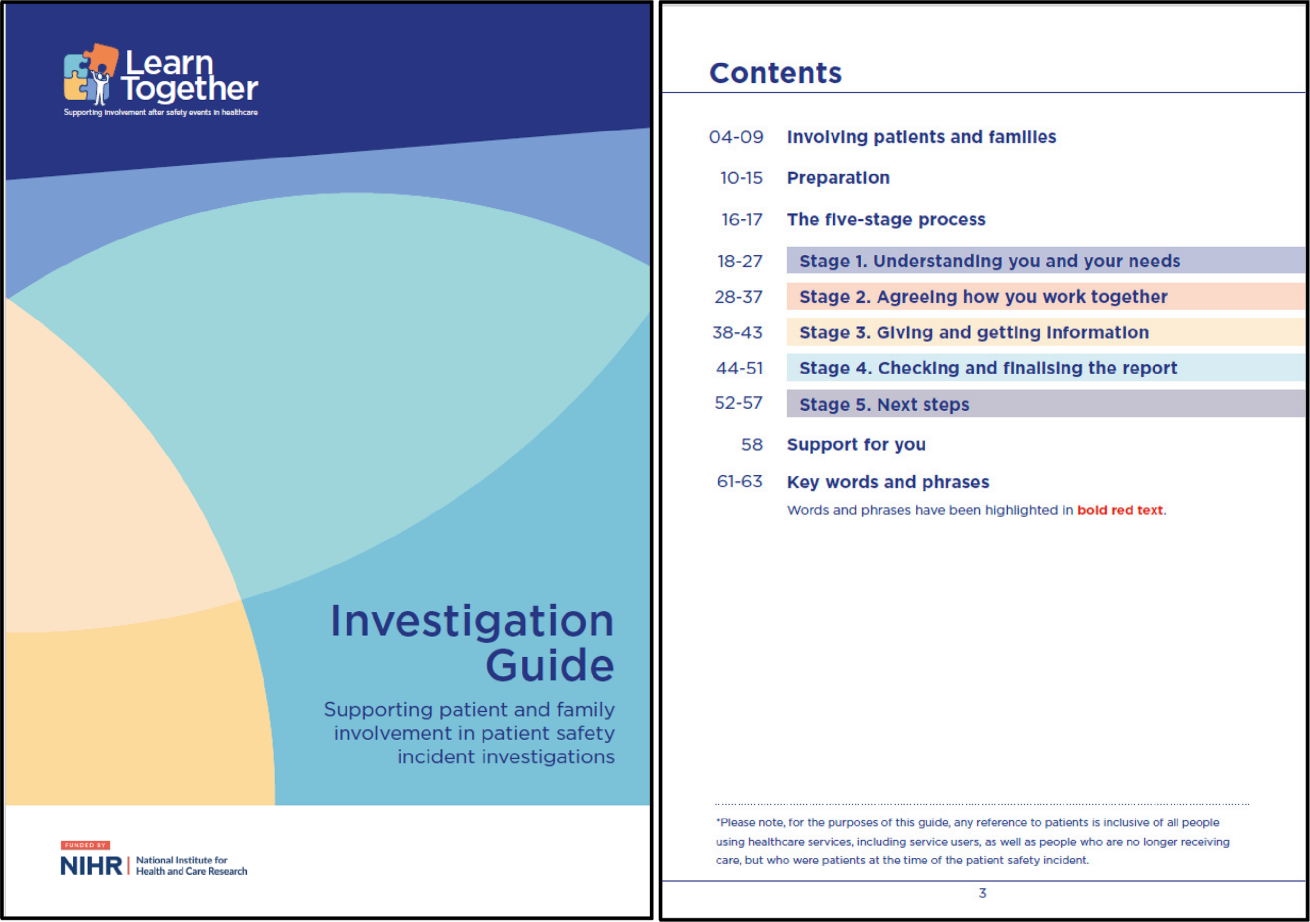
 *The underpinning five-stage Process:* 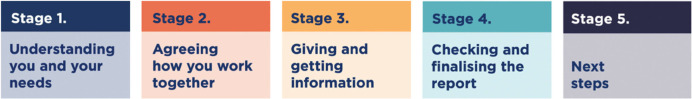

### 4.2 Strengths and limitations

The guidance has had a significant impact. It was shared at an initial stakeholder event including the co-design community and has since been signposted to from national guidance—PSIRF (15). To date, it has been downloaded by representation from >90% of NHS Trusts in England, and is being adapted for use in contexts locally, nationally and internationally. In terms of limitations, first, this study commenced while the effects of Covid-19 were felt, and as such we had to adapt some of our study design to accommodate social restrictions. Second, we were reliant on investigators to approach patients and families to take part in the study. We recognize that a lack of trust in staff working for the organization they experienced healthcare harm, at may mean that people chose not to take part. However, after consultation with a patient and family advisory group and a staff advisory group, we proceed in a way that on balance seemed most ethical. Nonetheless, there may have been important viewpoints lost due to sampling. Third, while we did not specifically collate demographic data, we recognize that our sample may have had limited diversity. This is an important issue, particularly as evidence is growing to suggest that people from underserved communities can be more likely to experience a safety event or poor-quality care [e.g., (41–46)]. To avoid exacerbating existing healthcare inequities, it is crucial that involvement is accessible and inclusive to all, with organizations adapting processes where possible, rather than simply expecting people to adapt. Therefore, we recommend that this is a future focus of research. Fourth, the findings may not be generalisable to other healthcare economies outside of NHS England.

## 5 Conclusions

Investigations of harm are complex, relational processes that have the potential to either repair, or compound harm. The Learn Together guidance helps to support the more systematic involvement of patients and families in investigations and informs people about how, why and when to become involved. This evaluation informed further revisions to the guidance, to better inform and support patients, families and investigators in ways that meet their needs (https://learn-together.org.uk). In particular, the five-stage process is designed to center the needs of patients and families to be heard, and their experiences dignified, before moving to address organizational needs for learning and improvement. However, given the highly complex, relational nature of investigations, our findings highlight a need for more formal recognition, support and training for the multi-faceted challenges investigators face. They also need to work with an organizational infrastructure which aligns with what they are trying to achieve and is flexible enough to allow them to individualize their approach. This is because it is unsustainable for individual investigators to absorb challenges alone and work against local and national systems to best support the meaningful involvement of patients and families after harm. Importantly, the healthcare system must be aligned in demonstrating that patient and family involvement is of real value, by adequately investing in, and resourcing activity that supports it and being flexible to accommodate to the needs of patients and families. Without that, the health service cannot move beyond being philosophically signed up to an idea underpinned by policy rhetoric, to achieve a workable, sustainable and system-embedded solution.

## Data Availability

A minimal data set can be made available by the corresponding author, upon reasonable request.
